# resVAE ensemble: Unsupervised identification of gene sets in multi-modal single-cell sequencing data using deep ensembles

**DOI:** 10.3389/fcell.2023.1091047

**Published:** 2023-02-15

**Authors:** Foo Wei Ten, Dongsheng Yuan, Nabil Jabareen, Yin Jun Phua, Roland Eils, Sören Lukassen, Christian Conrad

**Affiliations:** ^1^ Center for Digital Health, Berlin Institute of Health (BIH) at Charité—Universitatsmedizin Berlin, corporate member of Freie Universität Berlin, Humboldt-Universität zu Berlin, Berlin, Germany; ^2^ Charité - Universitätsmedizin Berlin, corporate member of Freie Universität Berlin, Humboldt-Universität zu Berlin, Department of Neurology with Experimental Neurology, Berlin, Germany; ^3^ Department of Computer Science, Tokyo Institute of Technology, Tokyo, Japan; ^4^ Health Data Science Unit, Faculty of Medicine, University of Heidelberg, Heidelberg, Germany

**Keywords:** bioinformatics, single-cell sequencing, deep learning, gene set analysis, rank aggregation, ensemble

## Abstract

Feature identification and manual inspection is currently still an integral part of biological data analysis in single-cell sequencing. Features such as expressed genes and open chromatin status are selectively studied in specific contexts, cell states or experimental conditions. While conventional analysis methods construct a relatively static view on gene candidates, artificial neural networks have been used to model their interactions after hierarchical gene regulatory networks. However, it is challenging to identify consistent features in this modeling process due to the inherently stochastic nature of these methods. Therefore, we propose using ensembles of autoencoders and subsequent rank aggregation to extract consensus features in a less biased manner. Here, we performed sequencing data analyses of different modalities either independently or simultaneously as well as with other analysis tools. Our resVAE ensemble method can successfully complement and find additional unbiased biological insights with minimal data processing or feature selection steps while giving a measurement of confidence, especially for models using stochastic or approximation algorithms. In addition, our method can also work with overlapping clustering identity assignment suitable for transitionary cell types or cell fates in comparison to most conventional tools.

## Introduction

A plethora of tools have been developed over the years for the analysis of high-throughput single-cell data (scRNA-seq, scATAC-seq, CITE-seq etc.) and made great contributions towards enabling the exploration and analysis of the omics landscape of different tissues and organs at an unprecedented resolution. Yet regardless of the tools or methods, human intervention is required to validate or further elucidate the hypotheses through the identification of markers or characteristic features (expressed genes, open chromatin peaks etc.) for the different cell populations clustered by cell type, perturbation, or other conditions and labels. Conventional methods and tools primarily perform highly variable genes (HVGs) or differentially expressed genes (DEGs) analyses based on the different clusters through statistical testing that often generate an extensive list of genes that are ranked according to their logarithmic fold change values or significance scores (Robinson et al., 2010; Love et al., 2014; Ritchie et al., 2015). These include tools such as Monocle (Cao et al., 2019), Scanpy (Wolf et al., 2018) and Seurat (Stuart et al., 2019) for scRNA-seq data, as well as ArchR (Granja et al., 2021), Signac (Stuart et al., 2021) and snapATAC (Fang et al., 2021) for scATAC-seq data. These lists are often cut on arbitrary values that are deemed meaningful in the different clusters within the studied biology, where the reduced lists of features still contain overwhelming numbers of genes even after manual curation. Removing outliers or specific features through prior knowledge could improve the analysis by restricting the scope at the cost of introducing certain biases. A complementary systematic approach could retain potentially interesting or novel features in a less biased manner.

These conventional analysis techniques backed by sound statistical tests and methods remain the gold standard for well-established tasks they excel at. However, machine or deep learning methods that can complement the nature of biological data are increasingly being applied as the complexity of these single-cell data increases. In contrast to statistical methods that look at genes in isolation, methodologies involving the use of deep learning techniques are context-aware and can deal with non-linearities. Moreover, as we gain access to new technologies and techniques that make new data collection possible, it is even more crucial to ensure that these data can be studied and analyzed using similar tools. In particular, the characteristics of variational autoencoders (VAE) make them suitable to address some of the most common tasks in analyzing single-cell sequencing datasets, especially when it involves the comparison or identification of cell populations (Way et al., 2020). A typical approach is to compare individual or a group of these cells and identify the features that characterize the populations. This can be performed by VAEs, where the model learns from the input data and identifies features that characterize the given annotations, which can consist of cell-state or cell-type identities, experimental batches, clinical metadata or perturbations etc. (Lotfollahi et al., 2019).

However, neural networks are non-deterministic during training, as they make use of random partitioning of data, initialization of weights, sampling in each iteration, regularization in terms of dropouts etc. (Scardapane and Wang, 2017). This randomness can contribute to robustness and improve the performance of these models, at the cost of making it difficult to identify consistent features as no two neural networks will be alike even if trained with the same parameters on the same data. Simpler models can usually reach convergence, but becomes challenging with more complex datasets or models, especially when the rankings of the outputs are taken into consideration. Thus, even if the identified features overlap across the different runs, their rankings are almost certainly not guaranteed to be consistent.

We previously introduced the restricted latent variational autoencoder (resVAE) neural network architecture that enables the decomposition of single-cell transcriptomic data into population-based features in a hierarchical manner that closely resemble biological systems (Lukassen et al., 2020). Our intention is to provide a supportive tool for additional knowledge mining on top or as a part of the user’s preferred analysis method. Here, we introduce resVAE ensemble with the use of rank aggregation to better address the consistency and confidence of feature identification in single-cell analysis. In contrast to conventional tools that focus on discrete cluster assignment, resVAE ensemble can be used on cell populations with transitory or partial cluster assignments. Most importantly, the introduction of deep ensembles allowed us to measure the consistencies or confidence metrics of the extracted features as determined by the different models.

## Results

**FIGURE 1:**
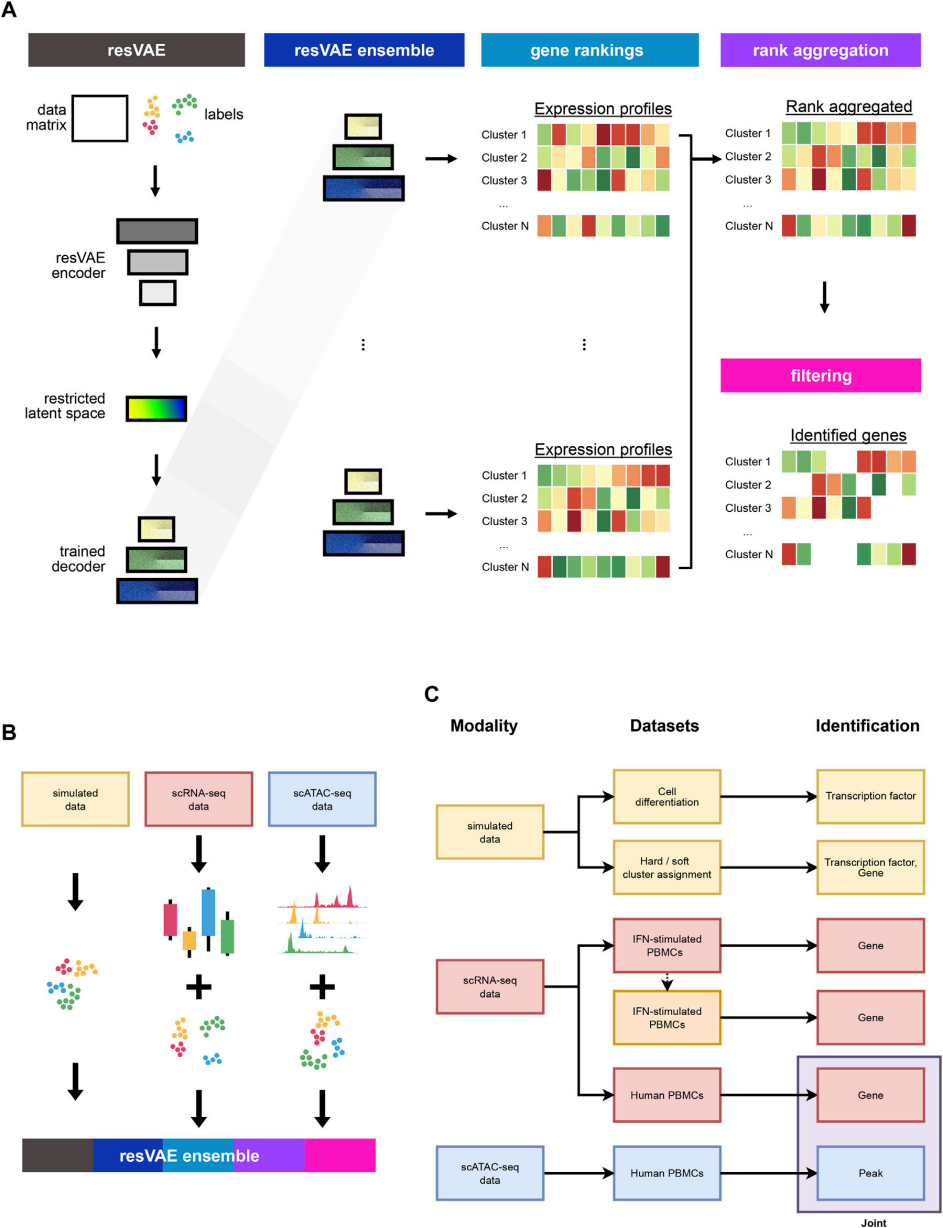
The variational autoencoder based resVAE architecture allows each label to have its own label-specific latent space for label-specific features identification. **(A)** shows the overall design of the resVAE ensemble workflow. **(B)** shows the use case of the resVAE ensemble methodology that can be applied to various forms of single-cell data with their corresponding cluster identities, including simulated data, scRNA-seq counts and scATAC-seq peaks. **(C)** highlights the overview of this manuscript. We highlighted the application of the resVAE ensemble methodology on simulated data and single-cell sequencing datasets of different modalities to identify features that could be used for further analysis.

With an ensemble, multiple models can be combined to produce better results than what an individual model alone could achieve. Meanwhile, rank aggregation allows us to retain the ranking information from the different ranked results and combine them into a single ranking. We trained multiple resVAE models on the same inputs to learn the cluster-specific features independently. Next, we combined the outputs of all the models and introduced different rank aggregation algorithms that could reduce them into a single ranked consensus, which results in more robust and consistent outputs as compared to using only a single model (Figure 1A). Furthermore, we also showed that resVAE ensemble can be applied on various single-cell data modalities such as simulated data, scRNA-seq transcript counts data and scATAC-seq peaks data to identity features for further analysis (Figures 1B, C).

### Advantages of resVAE ensemble on simulated dataset

To demonstrate that resVAE ensemble can identify features relevant to the experiment based on the input data and annotations, we first performed the analysis on a simulated synthetic dataset used to model myeloid differentiation in hematopoiesis as previously described by Krumsiek et al., 2011. In this dataset, common myeloid progenitors (Prog) are simulated to branch off into four different cell fates with distinct expression profiles, namely megakaryocytes (Mk), erythrocytes (Ery), granulocytes (Granu) and monocytes (Mono). In this simulated experiment, resVAE ensemble was performed on the obtained synthetic counts data and annotated labels to identify features that characterize the different cell fates.

**FIGURE 2:**
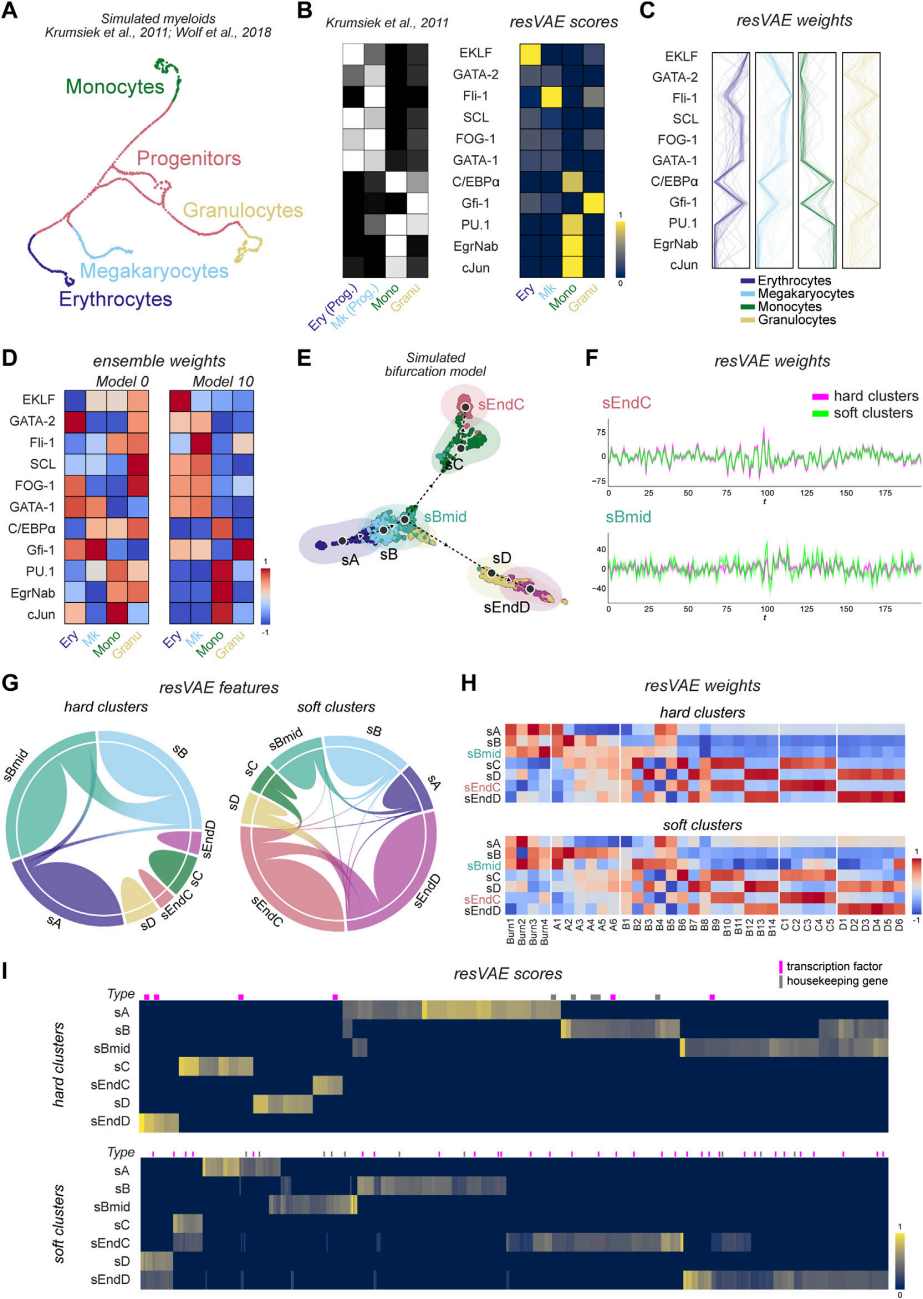
Results of resVAE ensemble on two simulated datasets. **(A**–**D)** correspond to the simulated PBMC dataset, while **(E**–**I)** correspond to the simulated bifurcation model dataset. **(A)** shows the UMAP of the cells from the simulated myeloid differentiation dataset provided in Scanpy (Krumsiek et al., 2011; Wolf et al., 2018). **(B)** shows the result of resVAE ensemble (right) in comparison the summarized expression levels of the 11 features present used to model the simulation (left, Krumsiek et al., 2011). **(C)** the lines show the weight mappings of these features in their corresponding clusters across all trained resVAE decoders. **(D)** shows the overall performance of two example models from the ensemble. (Ery: Erythrocytes; Mk: Megakaryocytes; Mono: Monocytes; Granu: Granulocytes; Prog: Progenitors). **(E)** shows the UMAP of the seven different cell states from the simulated bifurcation trajectory dataset. **(F)** shows the Andrews curves that highlight the structures of the resVAE decoders’ weights mappings of two different clustering analysis methods in two example populations, sEndC and sBmid. **(G)** shows the features identified by resVAE ensemble for the two different cluster assignment methods across all clusters. **(H)** shows the median weights mappings of the transcription factors across all models for the two cluster assignment methods. **(I)** shows the features identified by resVAE ensemble and their scores. The magenta and gray bars above the heatmaps correspond to transcription factors and housekeeping genes, respectively.

The UMAP of this data (Figure 2A) shows the different cell fates populations, where progenitors can be seen in the middle of the figure and branch off into the different main cell fates. In our findings, resVAE ensemble was able to identify the corresponding transcription factors that are relevant for each of these cell populations (Figure 2B). For instance, in the Erythrocytes and Megakaryocytes clusters, resVAE ensemble identified *EKLF* and Fli-1 as the defining feature for each of these clusters, respectively, as expected. Additional findings that conformed to experimental data were recapitulated too, such as GATA-1 being identified in both Erythrocytes and Megakaryocytes clusters. Similarly, features in the Monocytes cluster were identified consistently, with PU.1, cJun, EgrNab and C/EBP α standing out in comparison to the rest. In the Granulocytes cluster, resVAE ensemble identified Gfi-1 as the definitive feature, though it did not seem to consider PU.1 and C/EBP α . The consistency of the various trained models can be visualized using a parallel coordinate plot, where highly consistent clusters display thick uniform lines and *vice versa* (Figure 2C).

Additionally, to demonstrate the advantages of the ensemble, we also investigated the different resVAE models within the ensemble individually. While the resVAE model is quite robust in general, it is inevitable that models can get stuck at some local minima or overfit during training where major differences and variations between individual resVAE models can be observed (Figure 2D). On the one hand, we have Model 0 (Figure 2D, left) that did not manage to associate the *EKLF* feature with the Erythrocytes cluster as well as performing badly for the other clusters. On the other hand, Model 10 (Figure 2D, right) successfully identified relevant features for all the different clusters. With resVAE ensemble, these events can be detected and rescued by producing a consensus that showed reliable results (Figure 2B, right), where most of the identified features are valid and relevant, even if a few might be misidentified in some cases. Moreover, we also compared the performance of the different models trained with a combination of different hyperparameters (Supplementary Figure S1) in the Erythrocytes and Granulocytes clusters as an example. 10 individual models for each selected hyperparameters combination were trained separately to illustrate the differences that can arise from the training, even if the inputs and the hyperparameters are kept identical. In the Erythrocytes cluster, we observed that the different ReLU models tend to vary more, while models with a different activation function (Mish) hyperparameter are more consistent (Supplementary Figure S1). The Granulocytes cluster seems to pose a challenge for resVAE ensemble, as the different models display much more variety in comparison to the Erythrocytes cluster (Supplementary Figure S1). Interestingly, models with different hyperparameters seem to be able to identify different features better, for example the Mish activation function models identified *SCL*, FOG-1 and GATA-1 much more consistently in the Erythrocytes cluster and Gfi-1 in the Granulocytes cluster, in comparison to the ReLU models.

In summary, with this experiment we showed that resVAE can extract meaningful features from the simulated dataset, while the ensemble can aid in identifying and selecting features that are consistently identified. We also note that resVAE ensemble was able to identify these features without performing highly variable or differential expression comparisons.

### Advantages of resVAE ensemble that enable novel cluster assignment to cells on trajectory data with transitory cell populations

Next, we demonstrated a major advantage of resVAE ensemble that enables the use of soft or partial cluster assignments in addition to the hard or discrete cluster assignment that is performed routinely. Essentially, cells can be assigned partial identities consisting of mixed archetypes as would be expected in transitory cell states or developing cell types.

To demonstrate this, we constructed a synthetic trajectory data with interdependent hierarchical features simulating the roles of transcription factors-regulated gene expressions and investigated the performance of resVAE ensemble on this dataset (Supplementary Figure S2). In this bifurcation model, cells are simulated to transition from an initial state sA and fork into one of two distinct populations sC and sD under the influence of antagonistic regulators (Figure 2E). While a total of seven populations were defined in our simulation, we also show that it is possible to determine the optimal number of clusters using resVAE by measuring different clustering metrics (Supplementary Figure S3). Here, resVAE reported an optimal cluster number between 3 and 6 based on various scoring metrics, which seems reasonable based on the UMAP (Figure 2E). We then investigated and compared the performance of resVAE ensemble on two distinct (soft and hard) clustering and analysis methods.

Our findings in this simulated dataset again demonstrated that resVAE ensemble can identify population-specific features regardless of the cluster assignment methods. resVAE ensemble performed comparably using either method based on clustering evaluation metrics (Silhouette Coefficient of 0.52 vs. 0.55; Calinski-Harabasz score of 377.44 vs. 305.11; Davies-Bouldin score of 0.75 vs. 0.71). The main differences are most apparent in populations that are more heterogeneous and less consistent in terms of expression profile. This is most evident in sBmid which is not only very close and similar to sB, but would also incorporate similar cells belonging to the sC and sD populations in the soft clustering method (Figure 2E). In clusters that are more well defined such as sEndC, the structures of the trained resVAE decoders’ weights mappings are very consistent and highly overlapping regardless of the assignment method (Figure 2F, top). Meanwhile, the structure of the sBmid population is visibly disharmonized and misaligned in the models trained on the two different cluster assignment methods, indicating that the learned features are more different (Figure 2F, bottom). In the hard clustering-derived discrete clusters, these identified features are mostly exclusive to their own corresponding clusters, though some features are shared across clusters that are close to each other, as seen with clusters sA, sB and sBmid (Figure 2G, left). This is largely applicable to the soft clustering-derived clusters as well, though there are slightly more shared features identified due to the mixed or partial identities of some cells (Figure 2G, right).

In addition, resVAE ensemble successfully recapitulated meaningful results as defined in the transcription factors of the simulation (Figure 2H). For example, among the different module-specific transcription factors, the initialization of the sA state relies solely on the Burn1-4, B4 and B5 modules. This was captured strongly in the discrete cluster models (Figure 2H, top), as resVAE ensemble was able to identify the four Burn modules as well as the B4 and B5 modules specifically. Most of the differences in the soft cluster assignment method can be attributed to the fuzzier sB and sBmid populations (Figure 2H, bottom). The partial cluster models can better separate the effects of the A and B modules in the sBmid populations. Curiously, resVAE ensemble identified the D6 module in the sBmid population in the partial cluster models (Figure 2H, bottom), which could be explained by the proximity of a group of sD cells in the sBmid cluster. In the hard clustering method, this nuance is lost as these cells would not be included in the sBmid cluster. Regardless of the clustering methods used, the modules for the post-fork populations (sC, sD, sEndC, sEndD) were successfully identified, where the strong antagonizing interactions of these features can be observed. In both models, the B2, B6, B9, B10 and B11 modules that push sBmid to transition towards sC are identified in the sC and sEndC populations as expected. Similarly, the B3, B7, B12, B13 and B14 modules that push sBmid towards sD are likewise identified correctly. In addition, B8 that is present in both branches are identified, while terminal populations (sEndC and sEndD) tend to have stronger emphasis on their population-specific modules in comparison to the pre-terminal populations (sC and sD).

Regardless of the cluster assignment methods used, resVAE ensemble consistently identified not only features that can distinctly separate dissimilar populations, but also features that can be used to distinguish similar sub-clusters. Unsurprisingly, the features identified by resVAE ensemble can be affected by the fluidity of the clusters. In the case of hard clusters assignment, the identified features are more pronounced in the relevant clusters, whereas in the case of partial or mixed identities, the identified features are not as pronounced and tend to bleed into similar clusters (Figure 2I). Depending on the homo- or heterogeneity of the cells within their defined clusters, the number of features identified by resVAE ensemble could decrease or increase accordingly as the compositions of the clusters vary (Supplementary Figure S4).

In short, we successfully demonstrated that resVAE ensemble can be used with both discrete and mixed or partial cell identities that could characterize transitory cell types or cell states more accurately.

### Evaluating resVAE ensemble in real interferon β -stimulated PBMCs biological dataset

**FIGURE 3:**
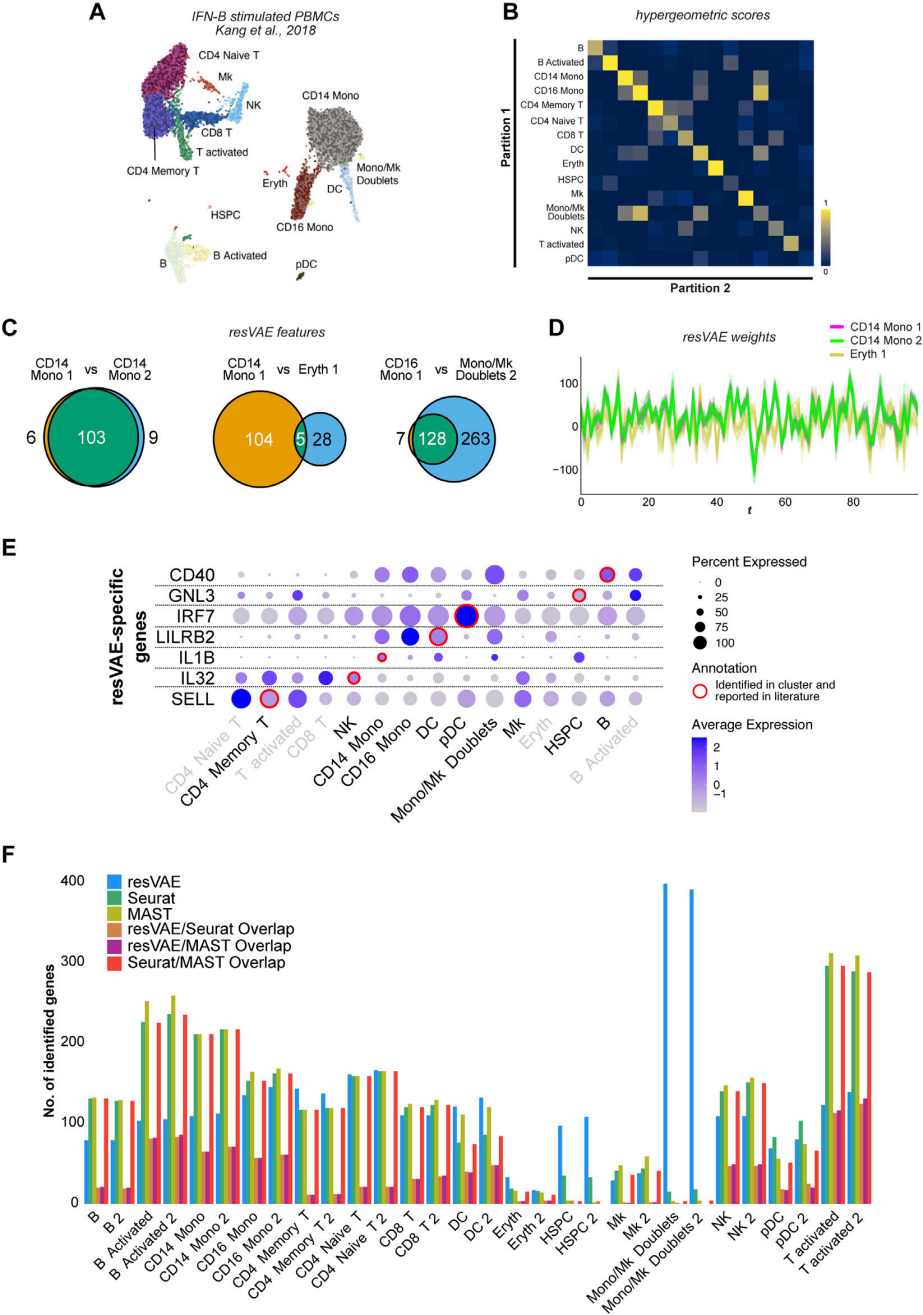
Results of explorative analyses using resVAE ensemble on the IFN-beta stimulated PBMC dataset. **(A)** shows the UMAP of the IFN-beta stimulated PBMC dataset with artificially introduced partitions shown in different shades. **(B)** shows the heatmap highlighting the significance scores of overlaps in genes identified by resVAE ensemble across the different clusters and partitions. **(C)** shows the number of overlapping identified genes between the different clusters. **(D)** shows the Andrews curves of the decoders’ median weights mappings described by the leftmost and middle Venn diagrams in **(C)**. **(E)** shows some selected examples of biologically meaningful genes missed by Seurat but identified by resVAE ensemble. **(F)** shows the comparison of the number of identified genes using resVAE ensemble, Seurat and MAST as well as how much they overlap.

To further investigate and demonstrate the performance of resVAE ensemble in gene sets identification tasks in actual biological data, we next applied the methodology on interferon (IFN) β -stimulated human peripheral blood mononuclear cells (PBMCs) scRNA-seq counts data (Figure 3A), which are mostly immune cells consisting of T cells, B cells, monocytes etc. (Kang et al., 2018).

To assess the performance of resVAE ensemble in elucidating cluster identities, we artificially split all the clusters into two partitions of equal sizes (Figure 3A). In total, 30 artificial cell type clusters were generated from 15 original cell type clusters. While not exactly comparable, under- or over-clustering occurs frequently during analysis depending on the granularity of the annotations. We systematically compared the different clusters and their identified genes by performing hypergeometric tests (Figure 3B). The results showed that neither the large numbers nor the similarities between the clusters affected the performance of resVAE ensemble.

In fact, we were able to identify consistent features that are relevant to the clusters. This is most prominent between the two partitions of the CD14 Mono cluster (Figure 3C, left) that highlighted an example of significant overlap with high scores, showing that artificially split clusters of the same origin not only yield highly overlapping identified genes in resVAE ensemble, but also relatively low or balanced non-overlapping genes. Here, resVAE ensemble identified 103 overlapping genes with only 15 genes that are not shared. These overlapping genes highly enrich for terms related to immune activities such as neutrophil activation, inflammatory response, cytokine-mediated signaling, cellular response to chemokine etc. This is exemplary considering the two partitions are not entirely identical and that some of the exclusive genes (*CD48*, *CTSC*, *CYBB*, *RTN4* etc.) have immune-related functions as well. Since the CD14 Monocytes constitute the largest population of cells in this dataset, resVAE ensemble was able to better learn the characteristics of these cells and identify genes more confidently.

Next, we also compared the genes identified by resVAE ensemble in the CD14 Monocytes 1 cluster *versus* the Erythrocytes 1 cluster (Figure 3C, middle), which is an example of two dissimilar clusters overlapping with a lower score and sparse number of overlapping ranked genes. Of the 137 genes involved, these two dissimilar clusters only share 5 overlapping identified ranked genes (*IER3*, *IL1B*, *LIMS1*, *NEAT1* and *TALDO1*). This contrast between almost identical (CD14 Mono 1 and CD14 Mono 2) *versus* highly dissimilar clusters (CD14 Mono 1 and Eryth 1) can be observed in the Andrews curves plot (Figure 3D), where the CD14 Mono 1 (magenta) and CD14 Mono 2 (lime) lines fully overlapped, *versus* the misalignment of the CD14 Mono 1 (magenta) and Eryth 1 (mustard) lines. Once again, this confirmed that resVAE ensemble can consistently distinguish between these different cell populations and identify genes that are relevant in the cluster-specific context.

Similarly, resVAE ensemble considers the CD16 Mono 1 cluster to be closely related to the Monocyte/Megakaryocyte Doublets 2 cluster, where almost all the genes identified in CD16 Monocytes are encompassed in the latter cluster (Figure 3C, right). Excluding these shared CD16 Monocytes cluster genes from the Monocyte/Megakaryocyte Doublets cluster yielded many genes that enrich for immune terms related to neutrophils, inflammatory responses, cytokines etc. Intriguingly, resVAE ensemble did not identify any shared or common genes between the actual Megakaryocytes cluster and the Monocyte/Megakaryocyte Doublets cluster (Supplementary Figure S5), suggesting that resVAE considers them as vastly different clusters which is also apparent from the UMAP plot (Figure 3A). However, some identified genes could be functionally linked to Megakaryocytes, such as platelet function (*CD9*, *FLNA*, *TLN1*, *PLA2G7*, *SERPING1*, *APLP2*, *ANXA5*, *MARCKS* etc.), cell adhesion (*THBS1*, *LGALSs*, *FERMT3* etc.), cell motility (*TPM4* etc.) and mediators of immune relevance (*IL1B*, *IL8*, *CXCL1*, *CXCL2*, *TGFBI* etc.) (Cunin and Nigrovic, 2019; Kammers et al., 2021). resVAE also managed to identify other biologically meaningful genes missed by Seurat, including a cell surface receptor on B cells involved in isotype switching (*CD40*; Bishop and Hostager, 2001), a cell adhesion molecule expressed on T cells that is a well-known marker for naïve and memory cells (*SELL*; Yang et al., 2011), a pro-inflammatory cytokine expressed on NK cells (*IL32*; Yang et al., 2019) and more (Figure 3E).

With this experiment, we further demonstrated that resVAE ensemble works on real scRNA-seq biological datasets, where the identified features correspond to the cluster identities in terms of biology and cell-type markers (Supplementary Figure S6). Clusters that are similar will have similar profiles and *vice versa*, thus potentially enabling a systematic approach to elucidate cluster identities. In addition, we showed that the performance of resVAE ensemble conforms to the clusters in a context-aware manner such that the number of clusters involved and their similarities did not seem to negatively affect the performance. resVAE not only identified genes consistently between the partitions, but also identified some genes that would be similarly identified using conventional methods such as Seurat and MAST (McDavid et al., 2015) as well (Figure 3F). Most importantly, we also showed that resVAE ensemble can capture biologically meaningful genes that could be missed by other methods (Supplementary Figure S7).

### Explorative analysis using resVAE ensemble on scRNA-seq and scATAC-seq multi-modal data

**FIGURE 4:**
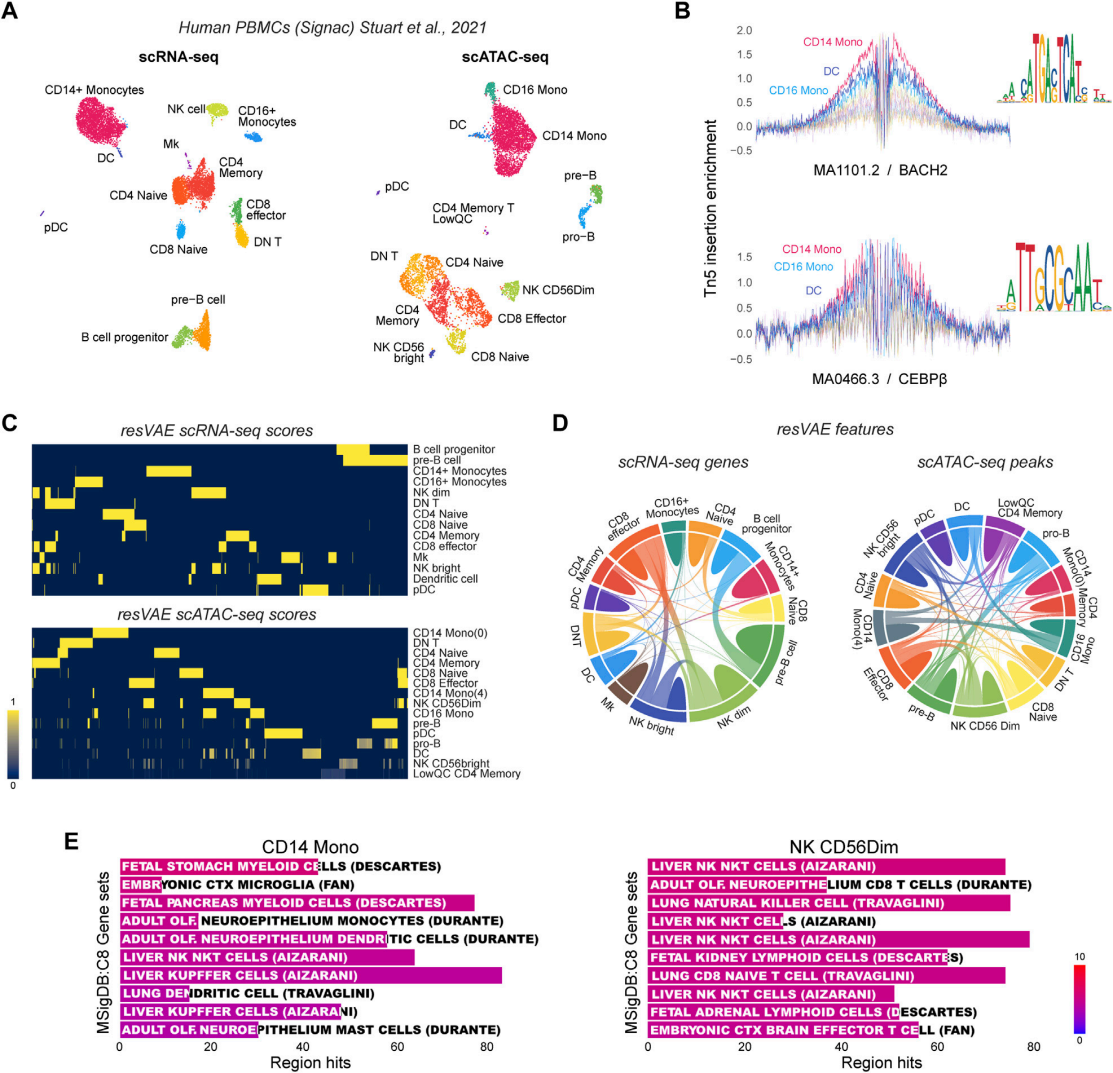
Results of explorative analyses using resVAE ensemble on the Human PBMC scRNA-seq and scATAC-seq datasets. **(A)** shows the UMAPs of the scRNA-seq and scATAC-seq datasets described by Stuart et al. (2021). **(B)** shows examples of transcription factor binding motifs and their footprinting identified in the Monocytes clusters. **(C)** shows the resVAE scores of the identified genes and peaks in the scRNA-seq and scATAC-seq data, respectively. **(D)** the chord diagrams highlight the extent of the sharing of identified features among the clusters. **(E)** shows examples of cell type enrichment terms that can be obtained from the identified peaks of CD14 Monocytes and NK CD56Dim clusters.

Next, we aimed to demonstrate that resVAE ensemble can be used directly on other single-cell data modalities such as scATAC-seq. To that end, we investigated the performance of resVAE ensemble on genes and peaks or open-chromatin region identification tasks in human PBMC scRNA-seq and scATAC-seq dataset respectively (Figure 4A). In contrast to single-cell RNA sequencing techniques that profile the transcriptomic landscape of single cells by capturing and measuring the RNA transcripts as a proxy to infer gene or protein expressions, single-cell ATAC sequencing techniques profile the epigenomic regulatory information of single-cells by capturing open regions in the chromatin accessible to regulators. One such possibility is to investigate transcription factor binding motifs that are enriched in these identified peaks. For instance, the MA1101.2/BACH2 and MA0466.3/CEBPβ motifs are examples where strong Tn5 insertion enrichment was observed, especially in the Monocytes and DCs clusters (Figure 4B).

In both scRNA-seq and scATAC-seq datasets, resVAE ensemble was able to consistently identify features that are relevant to the corresponding clusters (Figures 4C, D). The results also suggest that the identified genes and peaks are biologically meaningful, as clusters with similar identities or functions share more features in common and *vice versa*. For example, in the scRNA-seq dataset, the two B cells clusters—pre-B and B cell progenitor—have some identified features in common, while most of the T cells populations are also similarly clustered together based on the identified genes. Comparable results can be observed in the scATAC-seq dataset as well, where common peaks were identified in the Monocytes and B cells populations. Encouragingly, the two NK cells clusters were observed to share both genes and peaks not only between themselves but also with the CD8 Effector T cells that function similarly (Narni-Mancinelli et al., 2011) (Figures 4C, D).

Apart from common features, resVAE ensemble also identified distinct cluster-specific features, with the two CD14 Monocytes clusters being the most apparent considering the minimal connectivity between them (Figure 4C, bottom; Figure 4D, right). Meanwhile, a good balance between shared and distinct features was reached in the subpopulations of the B cells and NK cells clusters. In the case of scRNA-seq data, most clusters share a minimal number of genes, whereas clusters that do, share a considerable number of them, as shown among the NK cells and CD8 Effector T cells clusters, as well as the B cells clusters (Figure 4C, top). This is largely recapitulated in the scATAC-seq data where peaks were identified instead of genes (Figure 4C, bottom; Figure 4D, right). In addition, one of the CD14 Monocytes cluster seemed to share many peaks with the CD16 Monocytes cluster. Regardless, resVAE ensemble certainly identified more peaks that are shared in the scATAC-seq data in comparison to shared genes in the scRNA-seq data. In the biological context, the increase in shared features can be expected since different peaks could be mapped into one or several annotations and *vice versa*. Instead of identifying individual peaks that were considered distinct, the common genes that each distinct peak can be mapped to can now be identified and merged.

A brief cursory exploratory analysis into the enrichment results using the peaks identified in the NK CD56Dim cluster revealed some biologically meaningful results (Figure 4E). For instance, the peaks identified in both the CD14 Monocytes and NK cells clusters yielded enrichment terms that are highly suggestive of their cell type identities based on the Human Molecular Signature Database’s (MSigDB) “C8: cell type signature” gene sets (Liberzon et al., 2011). The identity of the NK cells cluster is obvious, while the identity of the Monocytes can be inferred after taking into consideration the samples studied (myeloid cells), and the non-monocytes terms such as microglia and Kupffer cells being closely related to monocytes or macrophages in terms of their function. Overall, the performance of resVAE ensemble here is within expectations, as PBMCs are remarkably similar in terms of their associated gene sets or enriched terms, not to mention the indirect interpretation at the open chromatin level.

Nevertheless, we successfully demonstrated the use of resVAE ensemble on joint or multi-modal analyses by analyzing scRNA-seq genes and scATAC-seq peaks of PBMCs with biological meaning. We also explored the regions identified by resVAE ensemble, which seemed to be relevant to the clusters in terms of the enriched transcription factor binding motifs or the closest genes that could be linked to these regions. In general, the consistencies of the identified features for specific clusters are affected by the number of cells in the cluster, where clusters with larger number of cells exhibit higher consistencies. We remark that we used the default hyperparameters for the resVAE models, even when the number of features in this scATAC-seq data with more than 87,560 peaks is more than 4.6-fold of the scRNA-seq data with 19,000 genes. Remarkably, the default encoder and decoder layers with 256 and 512 neurons seemed to be sufficient without having to scale up the model. These results also further suggested that the resVAE ensemble model is robust against hyperparameters tuning, making it feasible to get started immediately and have acceptable results to explore straightaway without excessive tweaking or benchmarking.

## Discussion

In this manuscript we detailed the introduction and implementation of a methodology combining deep ensembles with rank aggregation focusing on our resVAE architecture. resVAE ensemble is a methodology that implements rank aggregation of deep ensembles on the resVAE architecture that allows the capturing of cluster-specific hierarchical features in single-cell data. The introduction of deep ensembles with rank aggregation improves the consistencies and provides a measurement of confidence for the identified features. To be clear, we neither claim that resVAE ensemble itself is superior to other existing tools, nor intend for this to be a benchmarking study, since the methodology can be used alongside other reported methods. In addition, it is also not especially straightforward to directly compare and benchmark tools aimed at different use cases or niches without a good scoring metric or ground truths. This proves to be difficult when it involves context-specific biological interpretations, even more so without a strong confidence in terms of experimental or background knowledge. The aim of identifying relevant genes in a cell population also raises the question about how populations of mixed cell-types or cell doublets are treated, and whether they can be detected by resVAE ensemble. Our experiments showed that this may be achieved by splitting the cluster in question into several partitions and then comparing the identified genes among these partitions. The ratio of overlapping genes identified by resVAE ensemble between identical partitions should be much lower if the cluster consists of doublets or varied cell populations as demonstrated (Supplementary Figure S8).

Nevertheless, we observed that the features identified by resVAE ensemble generally resemble the HVG/DEGs from statistical testing-based methods like Seurat or Scanpy. However, the candidates identified by resVAE could be less biased in comparison to manual curation based on prior knowledge of what to expect, as they are not just restricted to the top genes of these methods. Here, we claim it could be less biased and interesting if we did not have to perform this curation at all, and let the network learn in a systematic manner instead.

The deep ensemble is most appropriate for methodologies utilizing approximation or probabilistic models that produce ranked results where the results are non-deterministic across different runs. Currently, ensembles of deep learning models are not commonly used. Even in classical ensembles such as random forest, the features are selected without taking rank aggregation into consideration. Here, with rank aggregation it is possible to measure the confidence or consistency of the identified features within the ensemble as we have shown. Alternatively, the deep ensemble can also be used to aggregate models with varying hyperparameters, especially when the best hyperparameters set are not known. Naturally, one can also perform equal or weighted aggregation of ensembles across methodologies to combine the results of different tools in a meaningful way.

Here we will also briefly discuss similar reported tools. The pathway module VAE (pmVAE) also utilizes VAEs in the form of pre-selected pathway modules that only act on the participating genes to encourage module independence (Gut et al., 2021). Hence, it can be used to determine the weights of each of these pre-specified pathway modules across each cell in the dataset. This is different by nature in comparison to resVAE that leverages any labeling information to retrieve features in a hierarchical manner. scArches is another family of tools with a bigger aim of tackling dataset integration by mapping queries to reference datasets (Lotfollahi et al., 2022). While it is possible to extract and cobble various parts of scArches together to achieve similar functionalities provided by resVAE, it neither addresses the same needs directly nor utilizes ensembles and rank aggregation.

Currently, clustering analysis is the standard in single-cell data analyses, where cells within the same cluster are treated in a homogenous manner. Archetypal analysis could be another option especially for transitionary cell types or states, where populations are described in terms of combinations of archetypes. We have shown that resVAE ensemble can be used in a similar fashion in the bifurcation model experiment, where populations were assigned by their similarities to specific archetypes. scAAnet is another tool that utilizes autoencoder for archetypal analysis by optimizing the archetypal constraint in the shared latent space (Wang and Zhao, 2022). Interestingly, the authors of scAAnet also remarked on the stochasticity of such deep learning methods. Moreover, they must train and evaluate multiple models manually to ensure that the outputs are stable. This is where we believe our approach with resVAE ensemble to be superior and should be highlighted, since we leveraged the inherent stochasticity by incorporating the consistencies and rankings of the outputs to yield more consistent results.

In terms of data analysis, we demonstrated that resVAE ensemble can be used on different single-cell data modalities, including scRNA-seq and scATAC-seq. In addition, we also showed that resVAE ensemble can be used to identify features for joint analysis of multi-modal data in combination with other tools. resVAE ensemble can further be used with cluster assignment methods that assign partial or mixed identities for the different clusters, which cannot be usually performed in most conventional tools used to analyze these data.

Overall, we introduced rank aggregation of ensembles that improved the consistency and reproducibility of the results. In fact, we believe that the ensemble and rank aggregation approach introduced can be applied to other similar methods to potentially yield better results. Moreover, it is possible to combine the results from resVAE with the results from other methodologies in the ensemble, whether they are widely used end-to-end analysis workflows or niche tools targeting specific use cases or even upcoming tools.

## Conclusion

Conventional methodologies used for single-cell data predominantly focus on comparing highly variable or differentially expressed features across different populations. Deep learning or approximation-based algorithms can be used to identify features in single-cell data in a hierarchical manner in line with biological systems. However, these methods can produce different results due to their inherent randomness. Here, we used deep ensembles on our resVAE architecture to improve the reliability and consistency of the results. Moreover, resVAE ensemble can give a measure of confidence or consistency for the identified features. Finally, this method can be used to integrate results from different methodologies and algorithms to produce a consensus result.

## Materials and methods

### resVAE ensemble

resVAE ensemble is built on top of the restricted latent variational autoencoder (resVAE), which we introduced in our earlier publication (Lukassen et al., 2020). Apart from the introduction of ensemble with rank aggregation, we also further optimized our resVAE architecture and implementation. The ensemble is constructed by combining multiple independent resVAE models trained using identical inputs with varying hyperparameters. The cost function of the resVAE ensemble network is inherited from the base resVAE architecture, with minor adjustments through the addition of two adjustable parameters α and β targeting the reconstruction loss and Kullback-Leibler (KL) divergence respectively:

$$L_{resVAE}=\alpha \sum (X_{input}-X_{recons}{)}^{2}+\beta \sum \frac{1}{2}(1+\log(\sigma )-(s-\mu {)}^{2}-e^{\log\left(\sigma \right)})$$ (1)

In short, the cost function of the resVAE architecture consists of two parts, namely the reconstruction loss and the KL-divergence, respectively. The reconstruction loss measures the mean-squared error between the original inputs and the outputs of the autoencoder, which in our case is usually the cell by feature matrix. The KL divergence is a measure to quantify the similarity between probability distributions. Here, σ and μ represent the variance and mean of the latent distribution, respectively, while s refers to the latent offset hyperparameter. The addition of the α term allows us to prioritize the reconstruction accuracy. Meanwhile, the β term transforms the regular VAE into a β -VAE, where the β parameter can be tweaked to encourage the model to balance between prioritizing latent disentanglement or reconstruction accuracy (Higgins et al., 2016). The β -VAE is identical to a normal VAE when β=α=1 , while higher β value would emphasize the statistical independence represented by the KL divergence rather than the reconstruction accuracy, and thus implicitly enforcing the de-correlation of the learned latent spaces. In general, we find that higher α or β can produce better results in terms of the identified features by providing a better balance between reconstruction accuracy and latent disentanglement or through a better loss landscape as shown in our experiments (Supplementary Figure S1). We also introduced the use of label smoothing for the one-hot encoded labels, which has been shown to improve model generalization and calibration in many state-of-the-art models and tasks by preventing the network from becoming over-confident (Müller et al., 2019).

Weights initialization methods can play a critical role in training neural networks to convergence (Kumar, 2017). While rectified linear unit (ReLU) seemed to make more sense in terms of how gene expression works in biological organisms, the gradient vanishing and/or exploding problem remains an issue when it comes to training neural networks. This problem can cause neurons to become inactive and never recover during training. In practice, this could contribute to a less stable result, making it difficult to both assess the performance of the models and to obtain systematic reproducible results (Lu et al., 2019). Here we settled on the adoption of the Mish activation function in the current version of resVAE ensemble. The Mish activation function has been benchmarked extensively to demonstrate considerable improvements pertaining training stability over ReLU and other activation functions in various models and tasks (Misra, 2019).

$$Mish=x\times \tanh\left(\ln\left(1+e^{x}\right)\right)$$ (2)

Apart from the implementation of new activation functions for the non-linearities, we also improvised the kernel initialization method of the dense layers based on the activation function used. Now, the He Uniform kernel initialization method is paired with ReLU activations, while the Xavier or Glorot Uniform method is paired with hyperbolic tangent-based activations, which should be a better pairing as demonstrated in multiple studies and experiments (He et al., 2015; Datta, 2020).

Moreover, we adopted the combination of Rectified Adam (RAdam) with the Lookahead mechanism as our optimizer. The original Adam optimizer with its adaptive learning rate is known to suffer from bad convergence problems especially during the early stage of training, where the variance is undesirably large due to the limited amount of training samples. RAdam attempts to address this by introducing a dynamic rectifier term that adjusts the adaptive momentum of Adam by stabilizing the variance of the adaptive learning rate while avoiding the need for manual warmup (Liu et al., 2019). Meanwhile, the Lookahead mechanism allows a faster convergence by interpolating between two sets of weights that are iteratively updated during the exploration and training process (Zhang et al., 2019). The combination of these two methods has been shown to improve the learning stability with minimal computation and memory costs, with the main benefit being its robustness without the need for further hyperparameters tweaking. Nevertheless, the training time and speed will be affected by varied factors, such as the size of the datasets, the hyperparameters used etc. Using the INF- β stimulated PBMC dataset as an example, one resVAE model took around 6 minutes to train on an NVIDIA A100, and we could train several of these models in parallel either on the same GPU or across multiple GPUs.

While we primarily demonstrated the use of ensemble on identical inputs with varying combinations of α and β , more complex hyperparameters combinations and ensemble setups are possible, especially in combination with outputs from different tools.

### Gene list cut-off

To further improve the gene candidates produced from the cut-offs in situations where it is difficult to identify definite elbow and knee points from the rank plots, a new cut-off calculation method is implemented. First, the data is split into N bins of equal sizes. In practice, we are usually interested in the point at which there is a pronounced drop (knee) or rise (elbow) in the weights, which are usually located in the first and the last bins, respectively. Hence, the knee point is calculated by connecting the median data point of the first bin to its first data point to obtain the rotation angle θ , such that the x -axis can be rotated to be parallel with this line and the point where y is maximum is used as the knee point. Conversely, the elbow point is calculated using the θ obtained by connecting the median data point of the $N^{th}$ bin to its last data point and taking the adjusted point where y is minimum. This new method allows better fine-tuning or control over the cut-offs, where the number of genes can be further increased or decreased where appropriate. The median of the different cut-offs is used as the cut-off point for the final aggregation for each label-specific list.

Here, we have an example where the new method would improve the performance by producing more consistent cut-offs and restricting the number of features selected (Supplementary Figure S9). In these cases, using the original method would yield less consistent cut-off points and result in the inclusion of excess features (Supplementary Figure S9). Here, the difference would be 3,000 extra genes candidates in the scRNA-seq dataset to more than 10,000 extra peaks in the scATAC-seq dataset.

### Rank aggregation

We implemented the Robust Ranking Aggregation (RRA) algorithm (Kolde et al., 2013) in Python for use with resVAE ensemble. In this manuscript, all rank aggregations are performed using the RRA algorithm to produce one consensus ranked feature list that is used for further analyses. The meta-analysis by information content (MAIC) algorithm that can aggregate ranked and unranked lists (Li et al., 2020) is implemented as well and can be used if the ranked lists should be assigned different weights.

### Data analysis

The human PBMC scRNA-seq and scATAC-seq datasets for the analysis were obtained from 10x Genomics as detailed in the Data Availability section. The myeloid differentiation simulated dataset is included in Scanpy (Krumsiek et al., 2011; Wolf et al., 2018), while the bifurcation model simulated data is generated using dyngen (Cannoodt et al., 2021). This simulation model consists of 7 cell populations with 35 transcription factors regulating 500 target genes and an additional 20 housekeeping genes that are not regulated by the transcription factors. The analyses were performed using different tools where they are applicable, including Seurat (Stuart et al., 2019), Scanpy (Wolf et al., 2018), Signac (Stuart et al., 2021) and resVAE (Lukassen et al., 2020) ensemble to assess and compare the results. Gene set enrichment analysis was performed using Metascape (Zhou et al., 2019) or Enrichr (Xie et al., 2021). scATAC-seq peaks were also analyzed using the GREAT algorithm (Tanigawa et al., 2022) *via* rGREAT (Gu, 2022), which assigns biological meaning to non-coding genomic regions by analyzing the annotations of the nearby genes.

## Data Availability

The original contributions presented in the study are included in the article/Supplementary Material, further inquiries can be directed to the corresponding authors. The code for our implementation of resVAE ensemble is available on GitHub (https://github.com/lab-conrad/resVAE-ensemble).

## Supplementary material

Supplementary Figure S1 Extracted weights mappings for the Erythrocytes and Granulocytes clusters from all resVAE decoders in the ensemble. 10 independent resVAE models are trained for each set of varying hyperparameters. ReLU and Mish are activation functions.

Supplementary Figure S2 Interactions of the different entities involved in the simulation. The simulation model consists of 7 cell populations with 35 transcription factors regulating 500 target genes and an additional 20 housekeeping genes that are not regulated by the transcription factors. The colored dots represent different transcription factors, while the genes they regulate are shown as connected gray dots. Housekeeping genes that are independent of this regulation are also shown as light gray dots towards the top left corner.

Supplementary Figure S3 Determining cluster number using resVAE. Various scores and errors of clustering metrics are shown, where the numbers above the bars indicate the number of clusters generated for the different resolutions. The Silhouette (higher better), Calinski-Harabasz (higher better) and Davies-Bouldin (lower better) scores are standard clustering metrics that measure how well-defined the clusters are, while the Dice errors (lower better) measure how similar the different clusters are in terms of overlapping features. The non-zero mean Dice errors include only groups with overlapping features for calculations. The general trends persist regardless of the beta value, with a higher beta emphasizing the de-correlation of the different clusters. The red arrowheads highlight the two best scores of the corresponding metrics.

Supplementary Figure S4 The chord diagram illustrates the differences and overlaps of identified features between the two different cluster assignment methods. The H and S prefixes correspond to the “hard” and “soft” cluster assignment methods, respectively.

Supplementary Figure S5 An overview of the genes identified by resVAE ensemble and the extent of their co-occurences across all clusters.

Supplementary Figure S6 Heatmaps of all resVAE-identified genes in the different clusters, highlighting that resVAE can identify population-characterizing genes instead of emphasizing cluster-specific or differentially expressed genes. The numbers at the bottom-left corners indicate the number of genes resVAE identified for that specific cluster. The ratios at the bottom-right corners indicate the number of cell-type specific markers from PanglaoDB (Franzén et al., 2019) identified by resVAE, against the total number of markers annotated in the database for that cluster. The colored bars above the columns indicate the corresponding clusters. Cell-types that are ambiguous, without resVAE-exclusive genes or absent from PanglaoDB are excluded.

Supplementary Figure S7 The complete dot plot showing the expressions of all PanglaoDB cell-type markers identified by resVAE but missed by Seurat. An outlined dot indicates that the gene is specified as a marker for the corresponding clusters (columns). The red outline additionally indicates that the gene has been reported to be relevant in the context of the corresponding clusters in existing literature. The Mono/Mk Doublets cluster is a special case where outlined genes were indeed reported to be relevant in either Monocytes or Megakaryocytes cell-type, but left without a red outline. Other cell-types that are ambiguous, without resVAE-exclusive genes or absent from PanglaoDB are excluded and marked in light gray.

Supplementary Figure S8 Identification of mixed cell-types or doublet clusters using resVAE ensemble. The top bars show the number of genes identified by resVAE ensemble for the different clusters. The bottom bars indicate the ratio of genes identified in common between the two partitions of the same cluster. Mixed cell-types clusters are synthetically generated by randomly sampling cells from the specified clusters and assigned the same label. UMI doublets are synthetically generated by randomly sampling two UMIs and summing up their counts. These synthetic clusters are then split into two partitions to compare their genes identified by resVAE ensemble. We observed that these synthetic clusters tend to exhibit a lower ratio of overlapping identified genes between the two partitions, especially if they consist of fuzzier or more heterogenous populations. Synthetic clusters are marked in black, the ambiguous Mk/Mono Doublets in dark gray, and the rest in light gray.

Supplementary Figure S9
**(A**, **B)** show the comparisons between the original and the improved cut-off calculation method in scRNA-seq and scATAC-seq data, respectively.

Supplementary Table S1 Links for data used in this study.

## References

[B1] Bishop G. A. Hostager B. S. (2001). Signaling by CD40 and its mimics in B cell activation. Immunol. Res. 24 (2), 97–109. 10.1385/IR:24:2:097 11594459

[B2] Cannoodt R. Saelens W. Deconinck L. Saeys Y. (2021). Spearheading future omics analyses using dyngen, a multi-modal simulator of single cells. Nat. Commun. 12 (1), 3942–3949. 10.1038/s41467-021-24152-2 34168133 PMC8225657

[B3] Cao J. Spielmann M. Qiu X. Huang X. Ibrahim D. M. Hill A. J. (2019). The single-cell transcriptional landscape of mammalian organogenesis. Nature 566 (7745), 496–502. 10.1038/s41586-019-0969-x 30787437 PMC6434952

[B4] Cunin P. Nigrovic P. A. (2019). Megakaryocytes as immune cells. J. Leukoc. Biol. 105 (6), 1111–1121. 10.1002/JLB.MR0718-261RR 30645026 PMC6984013

[B5] Datta L. (2020). A survey on activation functions and their relation with xavier and he normal initialization. *arXiv preprint arXiv:2004.06632* .

[B6] Fang R. Preissl S. Li Y. Hou X. Lucero J. Wang X. (2021). Comprehensive analysis of single cell ATAC-seq data with SnapATAC. Nat. Commun. 12 (1), 1337–1415. 10.1038/s41467-021-21583-9 33637727 PMC7910485

[B7] Franzén O. Gan L. M. Björkegren J. L. (2019). PanglaoDB: A web server for exploration of mouse and human single-cell RNA sequencing data. Database 2019, baz046. 10.1093/database/baz046 30951143 PMC6450036

[B8] Granja J. M. Corces M. R. Pierce S. E. Bagdatli S. T. Choudhry H. Chang H. Y. (2021). ArchR is a scalable software package for integrative single-cell chromatin accessibility analysis. Nat. Genet. 53 (3), 403–411. 10.1038/s41588-021-00790-6 33633365 PMC8012210

[B9] Gu Z. (2022). rGREAT: GREAT analysis - functional enrichment on genomic regions. Available at: https://github.com/jokergoo/rGREAT . 10.1093/bioinformatics/btac745 PMC9805586 36394265

[B10] Gut G. Stark S. G. Rätsch G. Davidson N. R. (2021). PmVAE: Learning interpretable single-cell representations with pathway modules. bioRxiv.

[B11] He K. Zhang X. Ren S. Sun J. (2015). “Delving deep into rectifiers: Surpassing human-level performance on imagenet classification,” Proceedings of the IEEE international conference on computer vision, Santiago, Chile, 07-13 December 2015 (IEEE), 1026–1034.

[B12] Higgins I. Matthey L. Pal A. Burgess C. Glorot X. Botvinick M. Lerchner A. (2016). beta-vae: Learning basic visual concepts with a constrained variational framework. bioRxiv.

[B13] Kammers K. Taub M. A. Mathias R. A. Yanek L. R. Kanchan K. Venkatraman V. (2021). Gene and protein expression in human megakaryocytes derived from induced pluripotent stem cells. J. Thrombosis Haemostasis 19 (7), 1783–1799. 10.1111/jth.15334 33829634

[B14] Kang H. M. Subramaniam M. Targ S. Nguyen M. Maliskova L. McCarthy E. (2018). Multiplexed droplet single-cell RNA-sequencing using natural genetic variation. Nat. Biotechnol. 36 (1), 89–94. 10.1038/nbt.4042 29227470 PMC5784859

[B15] Kolde R. Laur S. Adler P. Vilo J. (2013). RobustRankAggreg: Methods for robust rank aggregation. Bioinformatics 28 (4), 573–580. 10.1093/bioinformatics/btr709 PMC3278763 22247279

[B16] Krumsiek J. Marr C. Schroeder T. Theis F. J. (2011). Hierarchical differentiation of myeloid progenitors is encoded in the transcription factor network. PloS one 6 (8), e22649. 10.1371/journal.pone.0022649 21853041 PMC3154193

[B17] Kumar S. K. (2017). On weight initialization in deep neural networks. *arXiv preprint arXiv:1704.08863* .

[B18] Li B. Clohisey S. M. Chia B. S. Wang B. Cui A. Eisenhaure T. (2020). Genome-wide CRISPR screen identifies host dependency factors for influenza A virus infection. Nat. Commun. 11 (1), 164–218. 10.1038/s41467-019-13965-x 31919360 PMC6952391

[B19] Liberzon A. Subramanian A. Pinchback R. Thorvaldsdóttir H. Tamayo P. Mesirov J. P. (2011). Molecular signatures database (MSigDB) 3.0. Bioinformatics 27 (12), 1739–1740. 10.1093/bioinformatics/btr260 21546393 PMC3106198

[B20] Liu L. Jiang H. He P. Chen W. Liu X. Gao J. (2019). On the variance of the adaptive learning rate and beyond. *arXiv preprint arXiv:1908.03265* .

[B21] Lotfollahi M. Naghipourfar M. Luecken M. D. Khajavi M. Büttner M. Wagenstetter M. (2022). Mapping single-cell data to reference atlases by transfer learning. Nat. Biotechnol. 40 (1), 121–130. 10.1038/s41587-021-01001-7 34462589 PMC8763644

[B22] Lotfollahi M. Wolf F. A. Theis F. J. (2019). scGen predicts single-cell perturbation responses. Nat. methods 16 (8), 715–721. 10.1038/s41592-019-0494-8 31363220

[B23] Love M. I. Huber W. Anders S. (2014). Moderated estimation of fold change and dispersion for RNA-seq data with DESeq2. Genome Biol. 15 (12), 550–621. 10.1186/s13059-014-0550-8 25516281 PMC4302049

[B24] Lu L. Shin Y. Su Y. Karniadakis G. E. (2019). Dying relu and initialization: Theory and numerical examples. *arXiv preprint arXiv:1903.06733* .

[B25] Lukassen S. Ten F. W. Adam L. Eils R. Conrad C. (2020). Gene set inference from single-cell sequencing data using a hybrid of matrix factorization and variational autoencoders. Nat. Mach. Intell. 2 (12), 800–809. 10.1038/s42256-020-00269-9

[B26] McDavid A. Finak G. Yajima M. Deng J. Gersuk V. Shalek A. K. (2015). Mast: A flexible statistical framework for assessing transcriptional changes and characterizing heterogeneity in single-cell RNA sequencing data. Genome Biol. 16, 278. 10.1186/s13059-015-0844-5 26653891 PMC4676162

[B27] Misra D. (2019). Mish: A self regularized non-monotonic neural activation function. *arXiv preprint arXiv:1908.08681* .

[B28] Müller R. Kornblith S. Hinton G. E. (2019). When does label smoothing help? Adv. neural Inf. Process. Syst. 32, 02629. 10.48550/arXiv.1906.02629

[B29] Narni-Mancinelli E. Vivier E. Kerdiles Y. M. (2011). The ‘T-cell-ness' of NK cells: Unexpected similarities between NK cells and T cells. Int. Immunol. 23 (7), 427–431. 10.1093/intimm/dxr035 21665959

[B30] Ritchie M. E. Phipson B. Wu D. I. Hu Y. Law C. W. Shi W. (2015). Limma powers differential expression analyses for RNA-sequencing and microarray studies. Nucleic acids Res. 43 (7), e47. 10.1093/nar/gkv007 25605792 PMC4402510

[B31] Robinson M. D. McCarthy D. J. Smyth G. K. (2010). edgeR: a Bioconductor package for differential expression analysis of digital gene expression data. bioinformatics 26 (1), 139–140. 10.1093/bioinformatics/btp616 19910308 PMC2796818

[B32] Scardapane S. Wang D. (2017). Randomness in neural networks: An overview. Wiley Interdiscip. Rev. Data Min. Knowl. Discov. 7 (2), e1200. 10.1002/widm.1200

[B33] Stuart T. Butler A. Hoffman P. Hafemeister C. Papalexi E. Mauck W. M. III (2019). Comprehensive integration of single-cell data. Cell 177 (7), 1888–1902. 10.1016/j.cell.2019.05.031 31178118 PMC6687398

[B34] Stuart T. Srivastava A. Madad S. Lareau C. A. Satija R. (2021). Single-cell chromatin state analysis with Signac. Nat. methods 18 (11), 1333–1341. 10.1038/s41592-021-01282-5 34725479 PMC9255697

[B35] Tanigawa Y. Dyer E. S. Bejerano G. (2022). WhichTF is functionally important in your open chromatin data? PLOS Comput. Biol. 18 (8), e1010378. 10.1371/journal.pcbi.1010378 36040971 PMC9426921

[B36] Wang Y. Zhao H. (2022). Non-linear archetypal analysis of single-cell RNA-seq data by deep autoencoders. PLoS Comput. Biol. 18 (4), e1010025. 10.1371/journal.pcbi.1010025 35363784 PMC9007392

[B37] Way G. P. Zietz M. Rubinetti V. Himmelstein D. S. Greene C. S. (2020). Compressing gene expression data using multiple latent space dimensionalities learns complementary biological representations. Genome Biol. 21 (1), 109–127. 10.1186/s13059-020-02021-3 32393369 PMC7212571

[B38] Wolf F. A. Angerer P. Theis F. J. (2018). Scanpy: Large-scale single-cell gene expression data analysis. Genome Biol. 19 (1), 15–5. 10.1186/s13059-017-1382-0 29409532 PMC5802054

[B39] Xie Z. Bailey A. Kuleshov M. V. Clarke D. J. Evangelista J. E. Jenkins S. L. (2021). Gene set knowledge discovery with enrichr. Curr. Protoc. 1 (3), e90. 10.1002/cpz1.90 33780170 PMC8152575

[B40] Yang C. Siebert J. R. Burns R. Gerbec Z. J. Bonacci B. Rymaszewski A. (2019). Heterogeneity of human bone marrow and blood natural killer cells defined by single-cell transcriptome. Nat. Commun. 10 (1), 3931–4016. 10.1038/s41467-019-11947-7 31477722 PMC6718415

[B41] Yang S. Liu F. Wang Q. J. Rosenberg S. A. Morgan R. A. (2011). The shedding of CD62L (L-selectin) regulates the acquisition of lytic activity in human tumor reactive T lymphocytes. PloS one 6 (7), e22560. 10.1371/journal.pone.0022560 21829468 PMC3145643

[B42] Zhang M. Lucas J. Ba J. Hinton G. E. (2019). Lookahead optimizer: k steps forward, 1 step back. Adv. neural Inf. Process. Syst. 32, 1–10. 10.48550/arXiv.1907.08610

[B43] Zhou Y. Zhou B. Pache L. Chang M. Khodabakhshi A. H. Tanaseichuk O. (2019). Metascape provides a biologist-oriented resource for the analysis of systems-level datasets. Nat. Commun. 10 (1), 1523–1610. 10.1038/s41467-019-09234-6 30944313 PMC6447622

