# Supplementary figures and images for: resVAE ensemble: Unsupervised identification of gene sets in multi-modal single-cell sequencing data using deep ensembles
Source: Front Cell Dev Biol. 2023 Feb 15;11:1091047. doi: 10.3389/fcell.2023.1091047 (PMC9975353; doi:10.3389/fcell.2023.1091047)

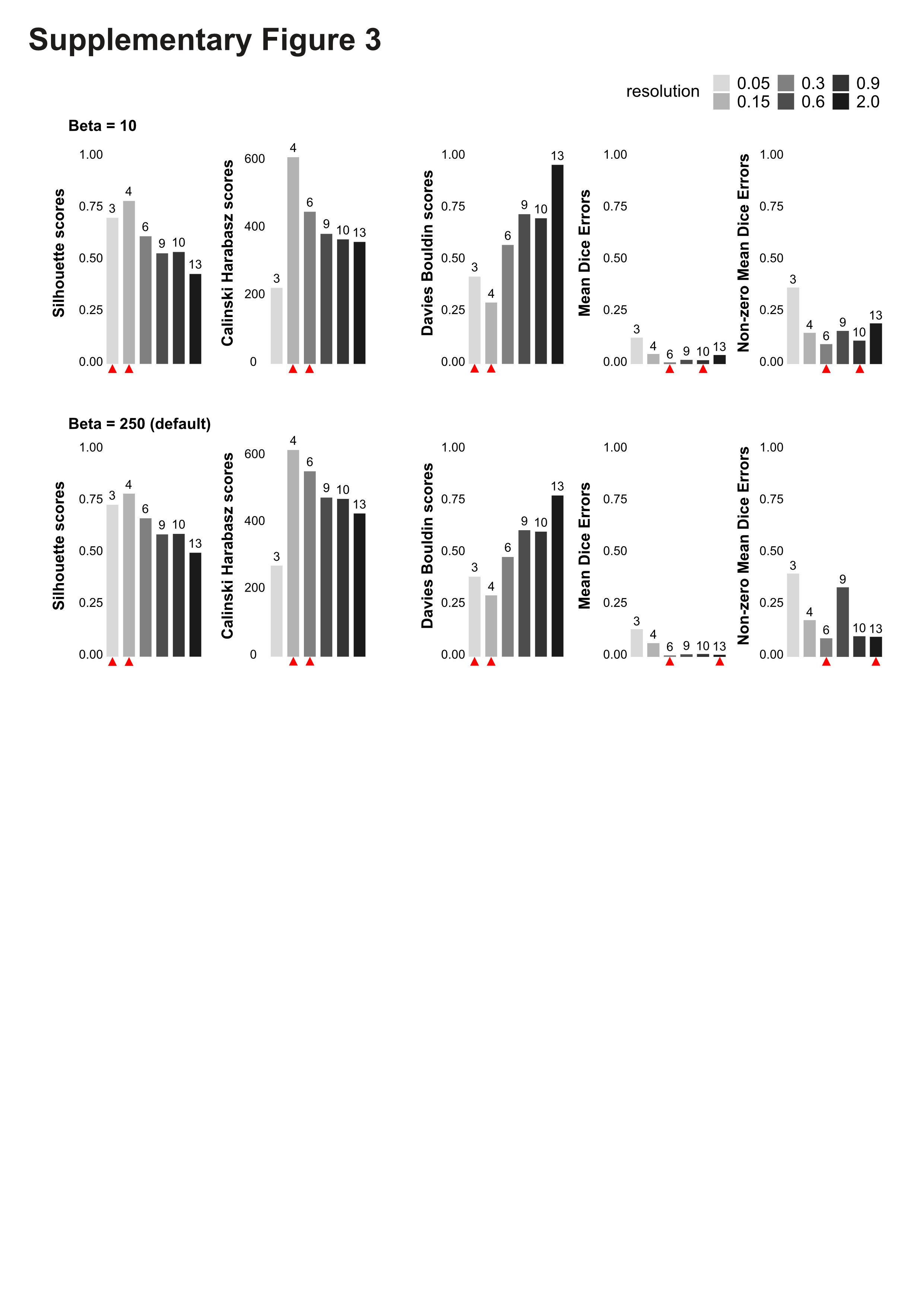

Supplement: Supplementary file 1 [file Image3.tiff]

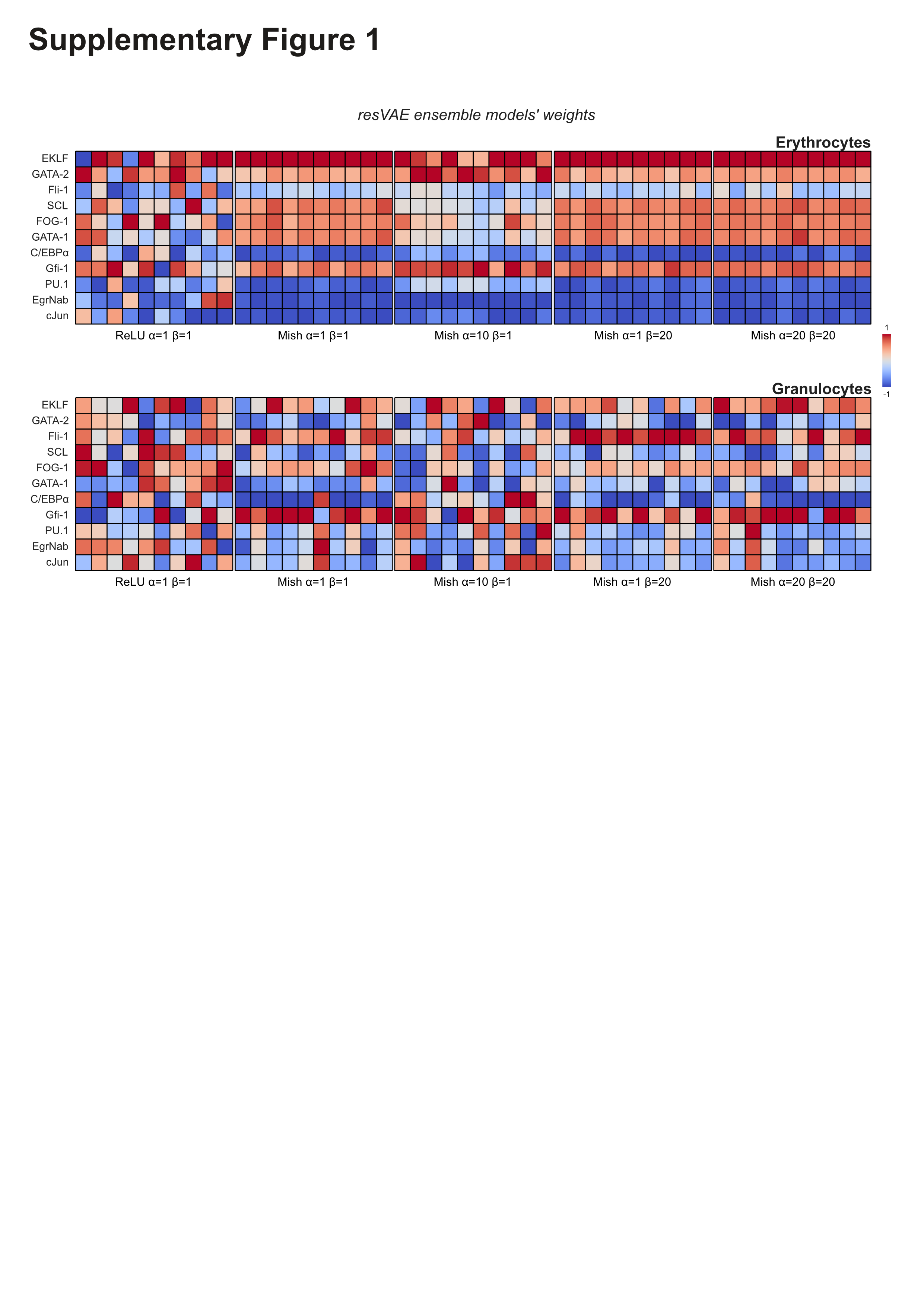

Supplement: Supplementary file 2 [file Image1.TIFF]

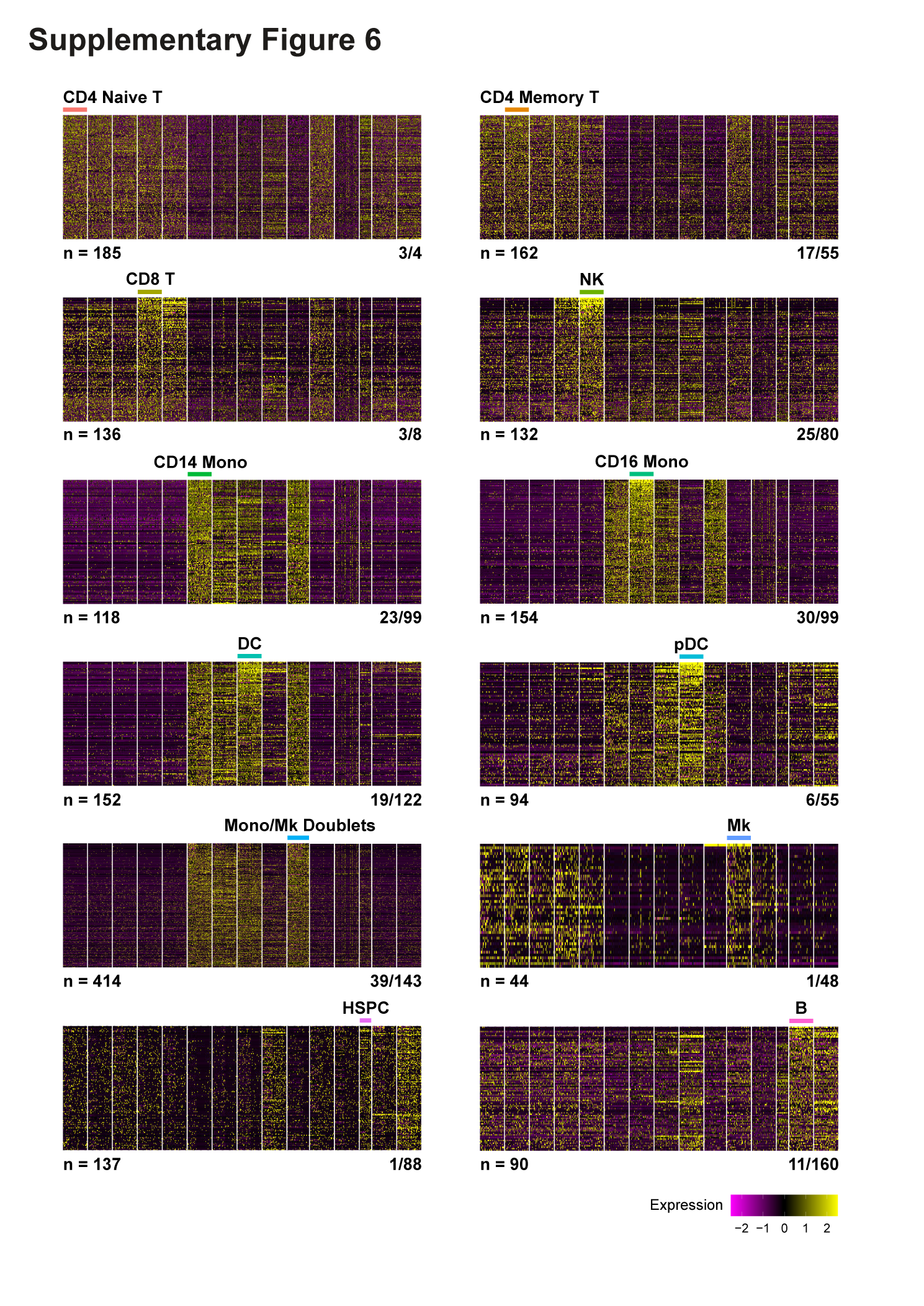

Supplement: Supplementary file 3 [file Image6.tif]

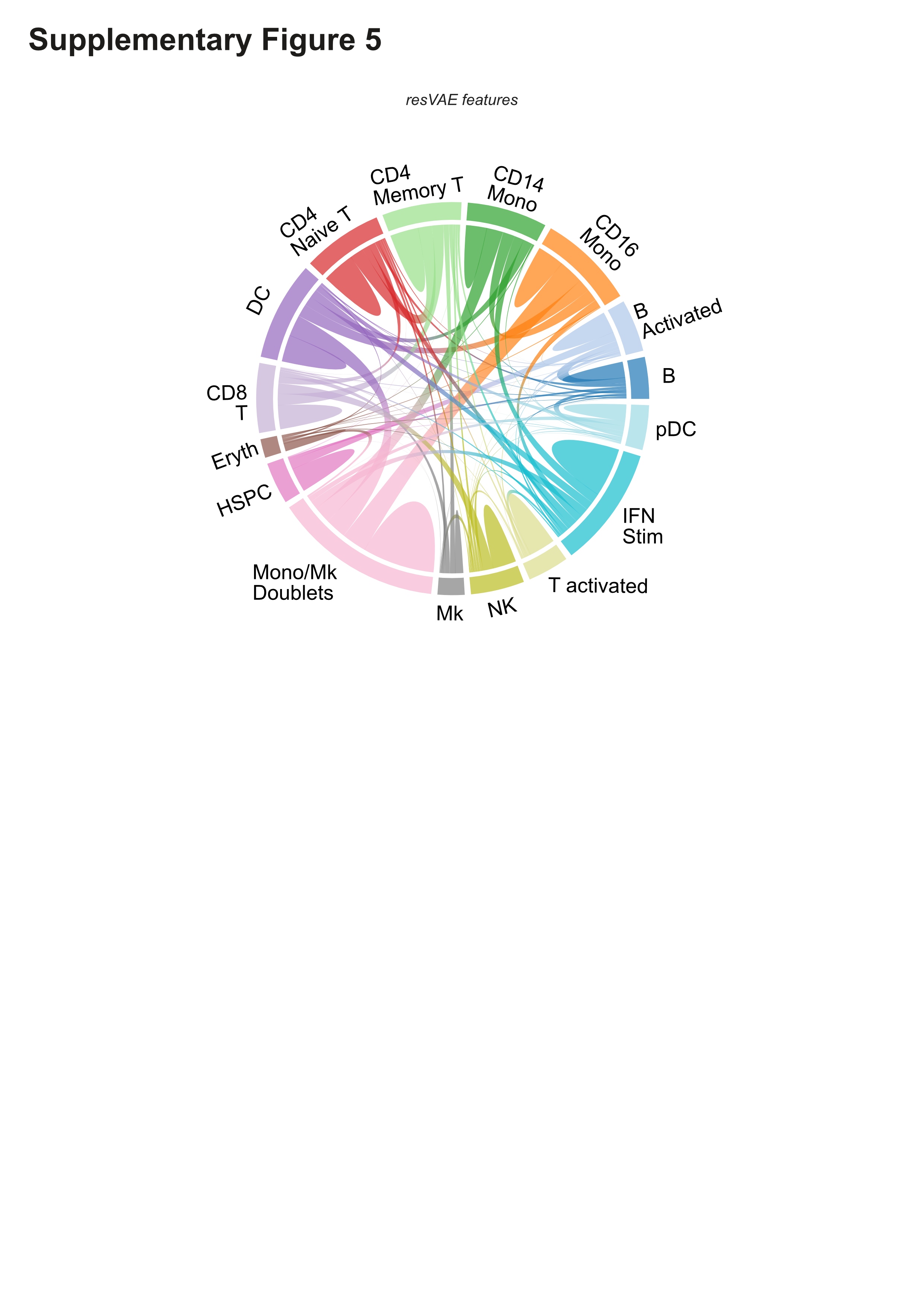

Supplement: Supplementary file 5 [file Image5.TIFF]

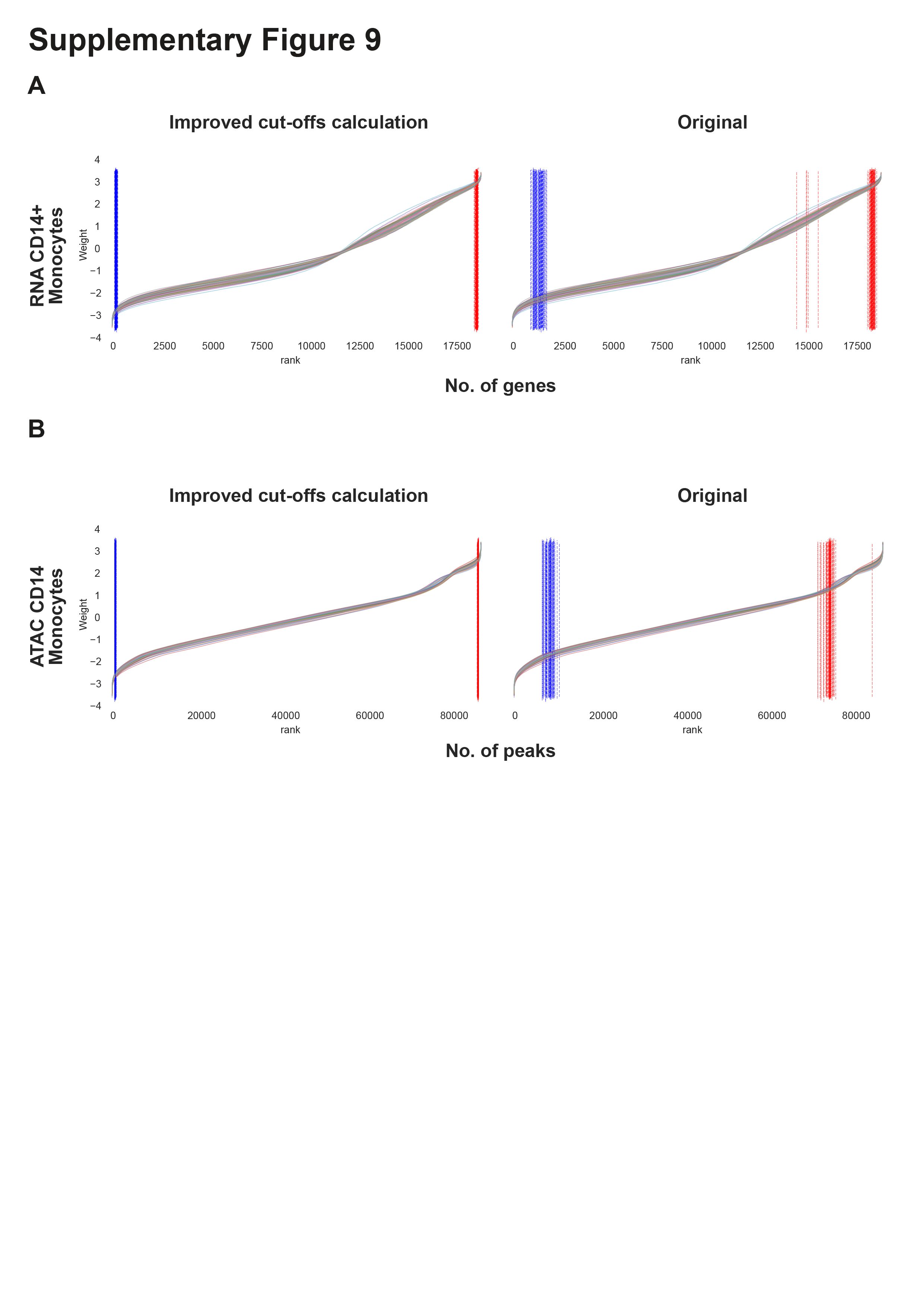

Supplement: Supplementary file 6 [file Image9.tif]

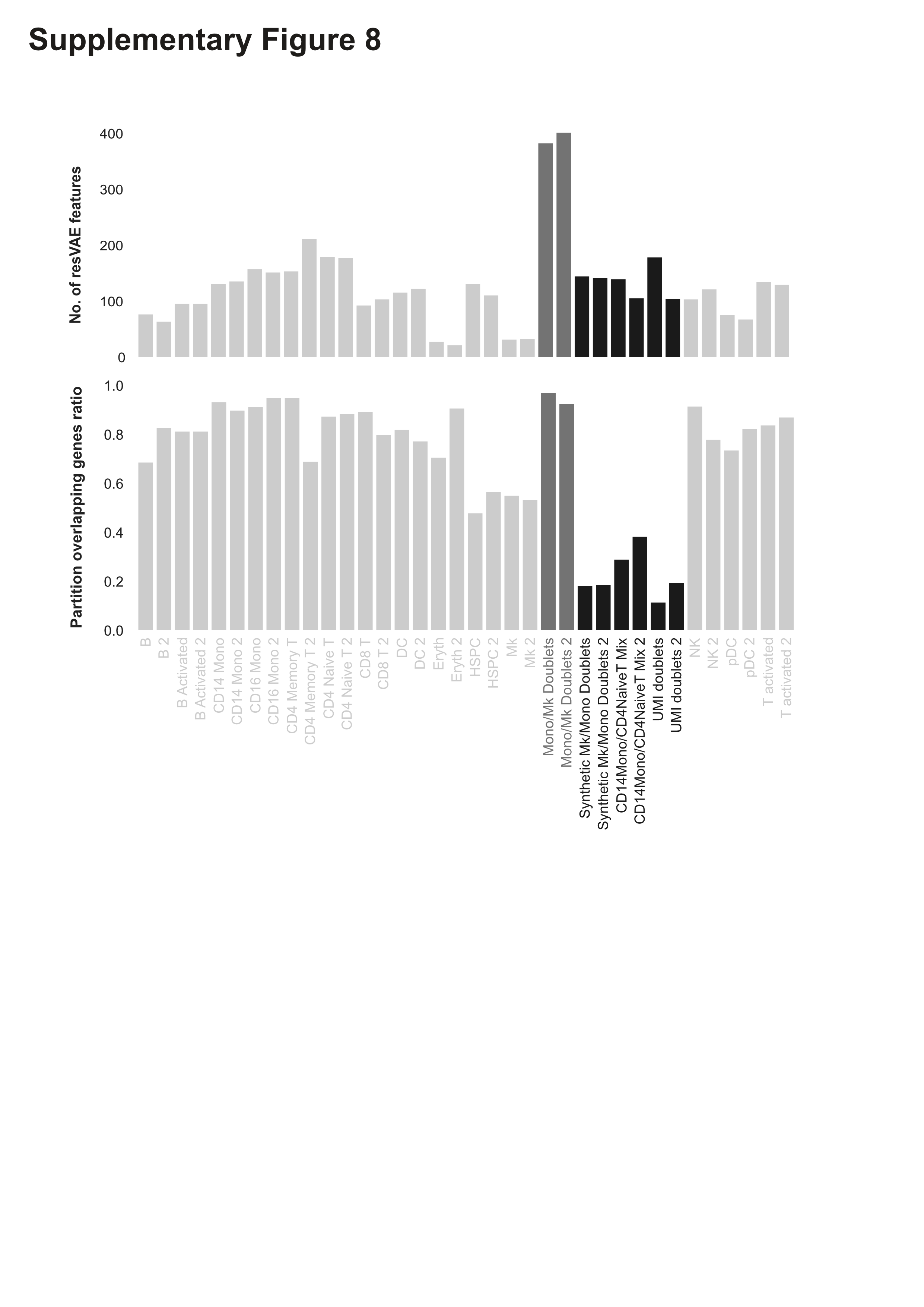

Supplement: Supplementary file 7 [file Image8.tiff]

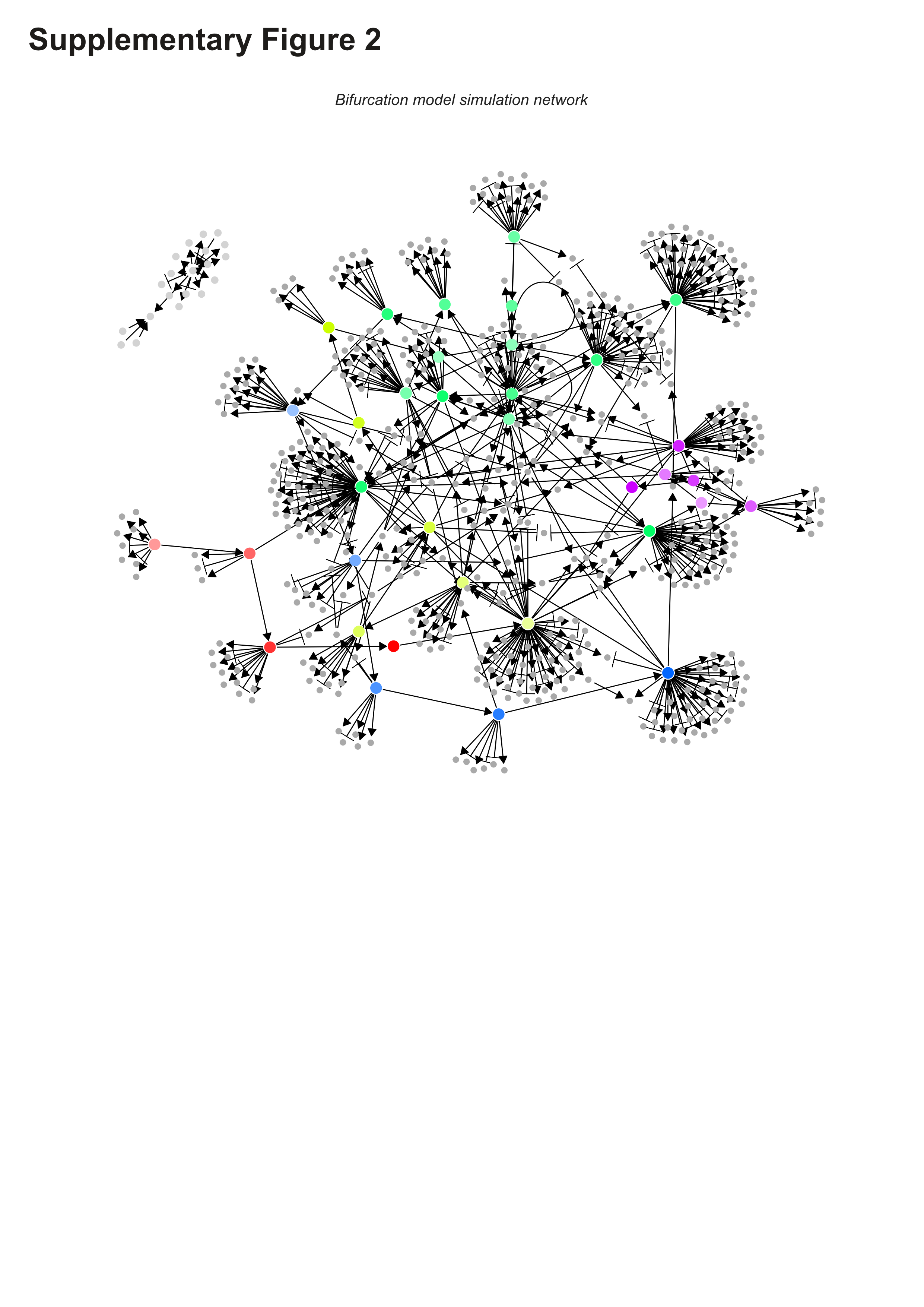

Supplement: Supplementary file 8 [file Image2.TIFF]

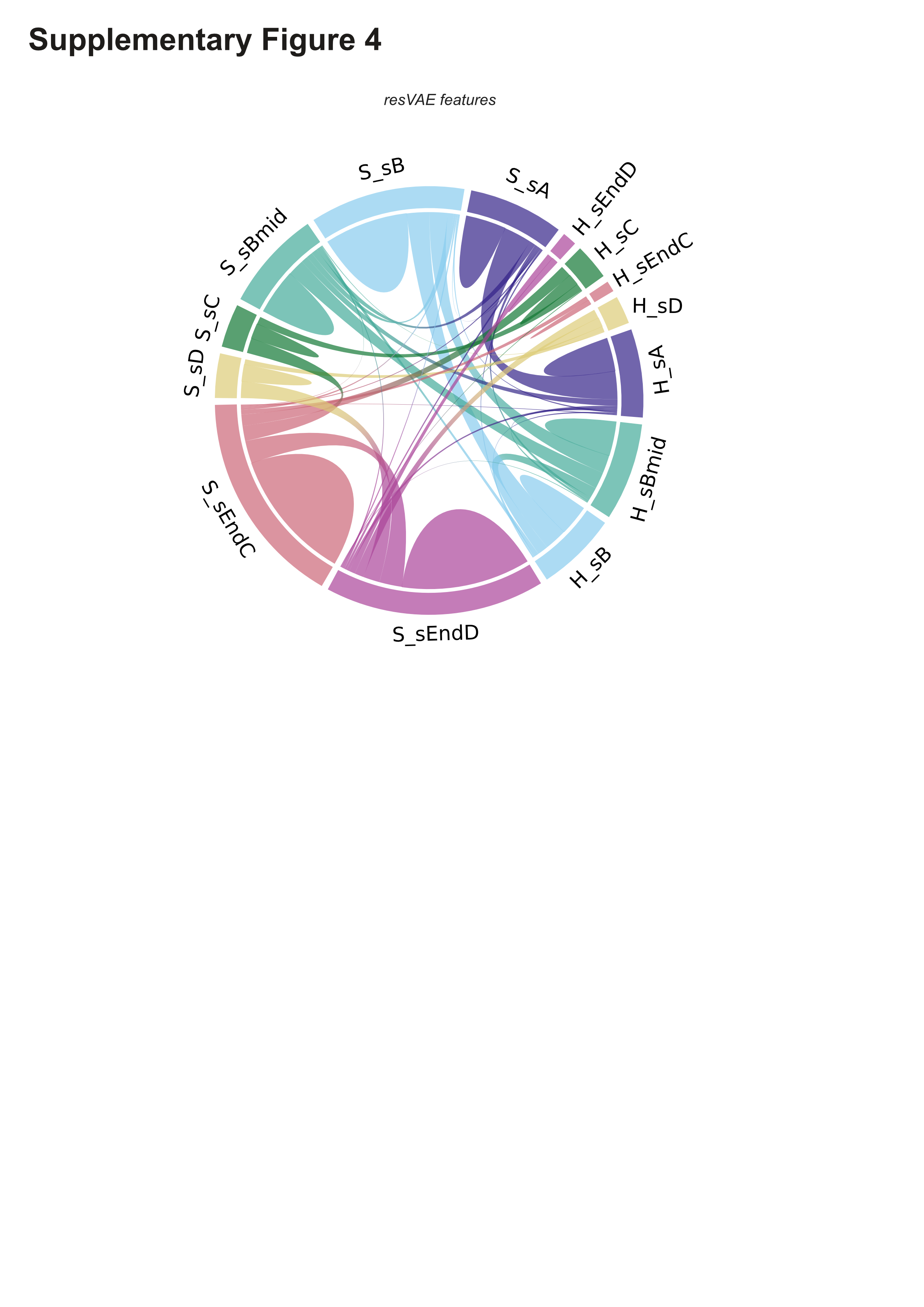

Supplement: Supplementary file 9 [file Image4.TIFF]

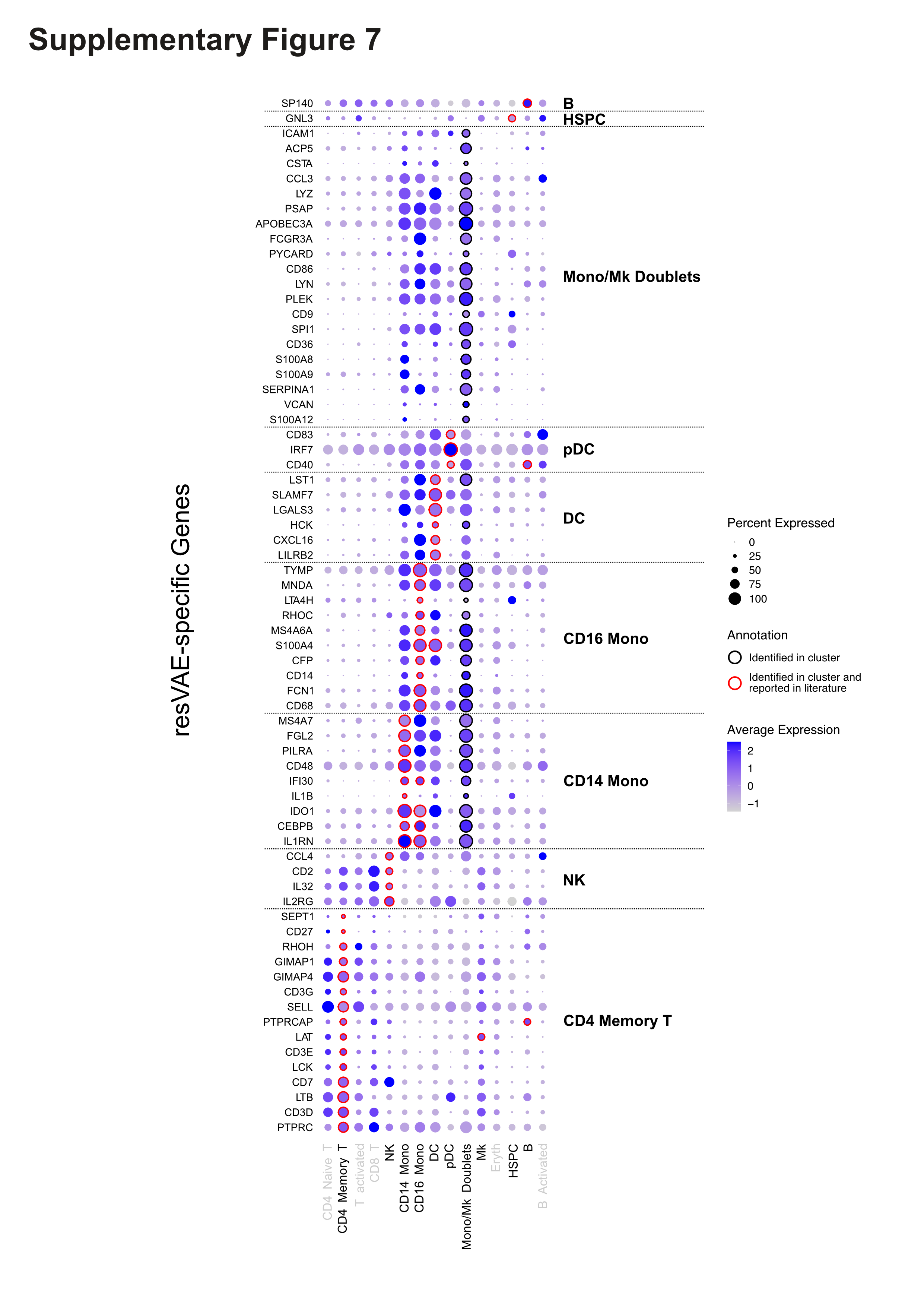

Supplement: Supplementary file 10 [file Image7.TIFF]
