# Supplementary material for: resVAE ensemble: Unsupervised identification of gene sets in multi-modal single-cell sequencing data using deep ensembles
Source: Front Cell Dev Biol. 2023 Feb 15;11:1091047. doi: 10.3389/fcell.2023.1091047 (PMC9975353; doi:10.3389/fcell.2023.1091047)
Supplement: Supplementary file 4 [file Table1.DOCX]

**Supplementary Table S1:** Links for data used in this study.
